# Comparison of the Antiproliferative Activity of Two Antitumour Ruthenium(III) Complexes With Their Apotransferrin and Transferrin-Bound Forms in a Human Colon Cancer Cell Line

**DOI:** 10.1155/MBD.1996.15

**Published:** 1996

**Authors:** F. Kratz, B. K. Keppler, M. Hartmann, L. Messori, M. R. Berger

**Affiliations:** 1 Tumour Biology Center Clinical Research Breisacher Straβe 117 Freiburg D-79106 Germany; 2 Anorganisch-Chemisches Institut der Universität Heidelberg Im Neuenheimer Feld 270 Heidelberg D-69120 Germany; 3 Laboratorio di Chimica Inorganica e Bioinorganica Universita degli Studi di Firenze Via G. Capponi 7 Firenze I-50121 Italy; 4 Institute of Toxicology and Chemotherapy German Cancer Research Center Im Neuenheimer Feld 280 Heidelberg D-69120 Germany

## Abstract

Two ruthenium(III) complexes, namely *trans*-indazolium[tetrachlorobis(indazole)- ruthenate(III)], HInd[RuInd_2_Cl_4_] and *trans*-imidazolium[tetrachlorobis(imidazole)- ruthenate(III)], HIm[RuIm_2_Cl_4_] exhibit high anticancer activity in an autochthonous colorectal carcinoma model in rats. Recently, it has been shown that both complexes bind specifically to human serum apotransferrin and the resulting adducts have been studied through spectroscopic and chromatographic techniques with the ultimate goal of preparing adducts with good selectivity for cancer cells due to the fact that tumour cells express high amounts of transferrin receptors on their cell surface.

In order to investigate whether the cellular uptake of the complexes was mediated by apotransferrin or transferrin, we compared the antiproliferative efficacy of HInd[RuInd_2_Cl_4_] and HIm[RuIm_2_Cl_4_] with its apotransferrin- and transferrin-bound form in the human colon cancer cell line SW707 using the microculture tetrazolium test (MTT).

Our results show that especially the transferrin-bound forms exhibit high antiproliferative activity, which exceeds that of the free complex, indicating that this protein can act as a carrier of the ruthenium complexes into the tumor cell.


### COMPARISON OF THE ANTIPROLIFERATIVE ACTIVITY OF TWO ANTITUMOUR RUTHENIUM(III) COMPLEXES WITH THEIR APOTRANSFERRIN AND TRANSFERRIN-BOUND FORMS IN A HUMAN COLON CANCER CELL LINE

F. Kratz*[1], B. K. Keppler[2], M. Hartmann[2], L. Messori[3], and M. R. Berger[4]

[1] Tumour Biology Center, Clinical Research, Breisacher Straße 117, D-79106 Freiburg, Germany

[2] Anorganisch-Chemisches Institut der Universität Heidelberg, Im Neuenheimer Feld 270, D-69120 Heidelberg, Germany

[3] Laboratorio di Chimica Inorganica e Bioinorganica, Universita degli Studi di Firenze, Via G. Capponi 7, I-50121 Firenze, Italy

[4] Institute of Toxicology and Chemotherapy, German Cancer Research Center, Im Neuenheimer Feld 280, D-69120 Heidelberg, Germany

### Abstract.

Two ruthenium(III) complexes, namely *trans*-indazolium[tetrachlorobis(indazole)-ruthenate(III)], HInd[$RuInd_2Cl_4$], and *trans*-imidazolium[tetrachlorobis(imidazole)-ruthenate(III)], HIm[$RuIm_2Cl_4$], exhibit high anticancer activity in an autochthonous colorectal carcinoma model in rats. Recently, it has been shown that both complexes bind specifically to human serum apotransferrin and the resulting adducts have been studied through spectroscopic and chromatographic techniques with the ultimate goal of preparing adducts with good selectivity for cancer cells due to the fact that tumour cells express high amounts of transferrin receptors on their cell surface.

In order to investigate whether the cellular uptake of the complexes was mediated by apotransferrin or transferrin, we compared the antiproliferative efficacy of HInd[$RuInd_2Cl_4$] and HIm[$RuIm_2Cl_4$] with its apotransferrin- and transferrin-bound form in the human colon cancer cell line SW707 using the microculture tetrazolium test (MTT). Our results show that especially the transferrin-bound forms exhibit high antiproliferative activity, which exceeds that of the free complex, indicating that this protein can act as a carrier of the ruthenium complexes into the tumor cell.

### Introduction

A number of Ru(III) complexes exhibit antitumour activity in animal models (Clarke, 1989). Keppler et al. have recently developed anticancer ruthenium(III) complexes with N-

* 1 Correspondence to F. Kratz, Tumour Biology Center, Clinical Research, Breisacher Straße 117, 79106 Freiburg, FRG

heterocycles, and, among these, the complexes with the ligands imidazole and indazole are the most promising (Keppler et al. 1989):

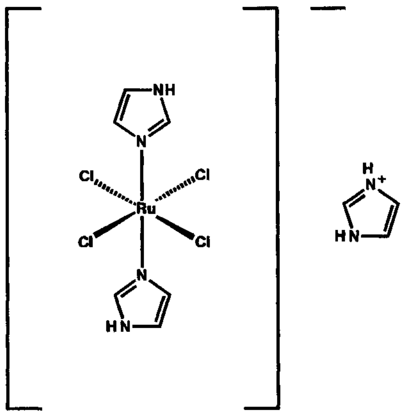

*trans*-Imidazolium-[tetrachlorobis(imidazole)-ruthenate(III), HIm[$RuIm_2Cl_4$]

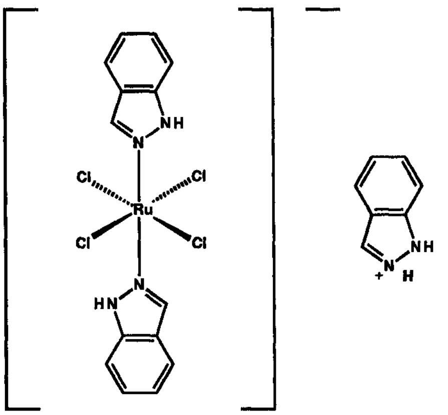

*trans*-Indazolium-[tetrachlorobis(2H-indazole)-ruthenate(III,N1), HInd[$RuInd_2Cl_4$]

These two complexes show high antitumour activity against an autochthonous colorectal cancer model in rats, a model which simulates the colon cancer of humans very well (Berger et al., 1989). Furthermore, both complexes exhibit antiproliferative activity in two human colon cancer cell lines (SW707 and SW948) (Galeano et al., 1992). Considering the observed $IC_{50}$ concentrations *in vitro* of 110 µg/ml for HInd[$RuInd_2Cl_4$] and 250 µg/ml for HIm[$RuIm_2Cl_4$] in SW707 with respect to possible tissue levels *in vivo*, it can be assumed from the dosage active *in vivo* of 13 mg/ml forHInd[$RuInd_2Cl_4$] and 7-10 mg/ml for HIm[$RuIm_2Cl_4$] that levels as high as the $IC_{50}$ value can be reached for a short period of time only or by tissue accumulation.

Since malignant cells have a high iron requirement and, consequently, a large number of receptors for the iron(III)-transport protein transferrin, it has been suggested that the accumulation of ruthenium(III) complexes in tumours might be mediated by this plasma protein (Clarke, 1989). Indeed, it has been previously reported that transferrin is responsible for the selective delivery of radioactive $^{67}$Ga(III) complexes to tumor tissues (Ward and Taylor, 1988). The significance of cellular uptake of transferrin with respect to various malignant diseases has been reviewed (Aulbert,1986).

Hence, to obtain a deeper insight of the interactions of antitumour Ru(III) complexes with serum proteins, we have recently investigated the reactions of HInd[$RuInd_2Cl_4$] and HIm[$RuIm_2Cl_4$] with human serum (Kratz et al., 1992 and 1993) and especially with apotransferrin (Kratz et al., 1994a): Protein-binding is rapid for HInd[$RuInd_2Cl_4$] (3-4 minutes at T = 37 °C) but takes about 5 hours for HIm[$RuIm_2Cl_4$], the major amount being bound to human serum albumin and a smaller fraction to human serum transferrin. Both complexes bind specifically around the iron(III) binding sites of apotransferrin as shown by circular dichroism spectroscopy. More precise information on the binding sites of the complexes has been revealed by soaking experiments of human apolactoferrin crystals with the two Ru(III) complexes. Preliminary results of the subsequent X-ray structure analyses have been reported (Kratz et al. 1994b) demonstrating that both complexes bind to the unoccupied binding site with the N-heterocycles remaining attached to the ruthenium[1].

Due to the protein-binding properties of these complexes, we wanted to investigate whether their cellular uptake was mediated by transferrin and therefore compared the antiproliferative efficacy of HInd[$RuInd_2Cl_4$] and HIm[$RuIm_2Cl_4$] with its apotransferrin- and transferrin-bound form in the human colon cancer cell line SW707 using the microculture tetrazolium test (MTT). The results of these experiments are reported in this communication.

### Materials and methods

*Substances and equipment.* The ruthenium(III) complexes were synthesized as described previously (Keppler et al., 1989). For the cell culture experiments the pure complexes were dissolved in 0.9% saline solution (c = 200 μg/ml). Human serum apotransferrin (98%, crystalline, essentially iron free, MW 80,000) (apoTf hereafter) and human serum transferrin (98%, crystalline, iron saturated, MW 80,000) were purchased from Serva, Heidelberg and Sigma Chemical Company respectively.

*Preparation of HInd[$RuInd_2Cl_4$] and HIm[$RuIm_2Cl_4$] bound to apotransferrin and transferrin respectively.* In the cell culture experiments varying amounts of the complexes (2, 4 and 8 equivalents) were bound to the proteins. The final concentration of the protein/complex solution was c = 200 μg of Ru(III)complex per ml.

In order to prepare 11.0 ml of the protein/complex solution with 2 equivalents of the complex, the following procedure was followed: 5.5 ml of an aqueous solution of the Ru(III) complex with c = 0.4 mg/ml (prepared by stirring at 37 °C in bidistilled $H_2O$ for 5-10 minutes) were added to a 5.5 ml solution of the protein (150.0 mg of apotransferrin or transferrin dissolved in the following buffer: 0.2 M NaCl, 0.008 M $NaH_2PO_4$, 0.05 M $NaHCO_3$) and then stirred for 1 hour in the case of HInd[$RuInd_2Cl_4$] (color of the solution changes from brown to green) and for 12 hours in the case of HIm[$RuIm_2Cl_4$] (color of the solution changes from orange to dark red). For binding 4 and 8 equivalents of the complex, 75.0 or 37.5 mg of either apotransferrin or transferrin were used.

Previous HPLC studies and ultrafiltration experiments have demonstrated that no free complex is present after the stated time periods (Kratz et al. 1992 and 1994). Thus, the protein/complex solutions were directly employed for the cell culture experiments.

Apotransferrin or transferrin running as control were dissolved in the same buffer as above and then diluted with bidistilled $H_2O$ (150.0, 75.0, or 37.5 mg protein dissolved in 5.5 ml buffer to which 5.5 ml bidistilled. $H_2O$ were added).

*Cell lines.* For the *in vitro* experiments a human cancer cell line SW707 was used, which was derived from patients with colorectal cancer and was provided by the Tumor Bank of the Institute of Experimental Pathology, German Cancer Research Center (Heidelberg, FRG). SW707 is a cell line derived from well-differentiated adenocarcinomas of the rectum with a modal chromosome number of 47. Microscopically, SW707 showed no microvesicular bodies. The cell line was routinely checked for their mucus production to exclude the possibility that fibroblasts had overgrown the carcinoma cells. Moreover, they were tested for

---

[1] The manuscript giving details of the X-ray structure analysis is in preparation (Baker et al.)

mycoplasma contamination and proved to be negative. The cell lines were grown as monolayer cultures in MEM medium (Gibco) supplemented with 10% heat-inactivated (57 °C, 40 · min) fetal calf serum, streptomycin (100 µg/ml), penicillin (100 IU/ml) and L-glutamine (2 µmol/ml; all from Serva, Heidelberg, FRG). The cells were maintained in a humified atmosphere (5% $CO_2$/95% air) at 37 °C.

*MTT test.* Monolayer cell cultures were trypsinised and single-cell suspensions were obtained by repeated pipetting. The percentage of viability was determined by the trypan-blue exclusion test. A final concentration of approximately 2 x $10^4$ cells/ml medium was prepared, and then 1.0 ml was added to each well of a 24-well culture plate. On the day following the plating of cells, the test compounds were added at the respective concentrations. The MTT assay was performed after the cells had been incubated with the test substances for 3 consecutive days (days 2, 3 and 4).

3-(4,5-dimethylthiazol-2-yl)-2,5-diphenyltetrazolium bromide (MTT; Sigma, FRG) was dissolved in phosphate-buffered saline (Oxoid Ltd., Basingstoke, England) at 5 mg/ml and filtered through a 0.22-µm Millipore filter, after which 100 µl of this MTT solution was added to each well (0.1 ml/ml medium). After incubation of the plates with MTT for 1 h at 37 °C, the medium was discarded and 500 µl acid-isopropanol (0.2 ml 0.04N HCl in 10 ml isopropanol) was added to all wells to stop the enzyme reaction. Within 1 h of this addition the extinction of the colored formazon derivative dissolved in acid-isopropanol was determined on a flow Flow Multiscan MC plate reader at a wavelength of 540 nm (reference wavelength: 690 nm).

At a given concentration, the MTT test was performend in 4 wells and the mean value calculated from these experiments is presented in Figures 1 and 2. Standard deviations were below 15% and are not shown in the figures.

### Results

The results of our cell culture experiments are shown in Figures 1A and B for the $HInd[RuInd_2Cl_4]$ series and in Figures 2A and B for the $HIm[RuIm_2Cl_4]$ series. 2, 4 and 8 equivalents of the Ru(III) complex were bound to the proteins in order to establish whether varying amounts of the bound complex had an effect on antiproliferative efficacy. The extinction values are displayed as columns for days 2, 3, and 4, and the tested concentrations were 100, 50 and 25 µg/ml of the free or protein-bound complex.

Figure 1A illustrates that the transferrin-bound forms of $HInd[RuInd_2Cl_4]$ exhibit a strong inhibitory effect at all of the concentrations tested when compared to the control and exceeds that of the free complex at the low dose of 25 µg/ml. By contrast, transferrin alone exhibits a strong stimulatory effect at high concentrations, and this result was to be expected considering that transferrin is an essential growth factor for cell culture.

The apotransferrin-bound form of $HInd[RuInd_2Cl_4]$ is not as active as the transferrin-bound form as can be seen in Figure 1B. Also, the free complex exhibits a strongerinhibitory effect than the apotransferrin-bound forms. Nevertheless, the protein-bound form exhibits quite a strong inhibitory effect at the high concentration of 100 µg/ml compared to the control and exceeds that of pure apotransferrin, which also shows an inhibitory effect compared to the control.

**Figure 1A: Comparison of the inhibitory effect of the free complex HInd[$RuInd_2Cl_4$] and its $Fe(III)_2$-transferrin-bound forms in the human colon cancer cell line SW707 using the microculture tetrazolium test (MTT).**

Different amounts of HInd[$RuInd_2Cl_4$] (2,4 and 8 equivalents) were bound to $Fe(III)_2$-transferrin. 100, 50, 25 represent 100, 50 or 25 g/ml of HInd[$RuInd_2Cl_4$]. Fe100, Fe50, Fe25 are comparative amounts of pure $Fe(III)_2$-transferrin corresponding to the amounts of the protein used to prepare the protein-bound forms of HInd[$RuInd_2Cl_4$].

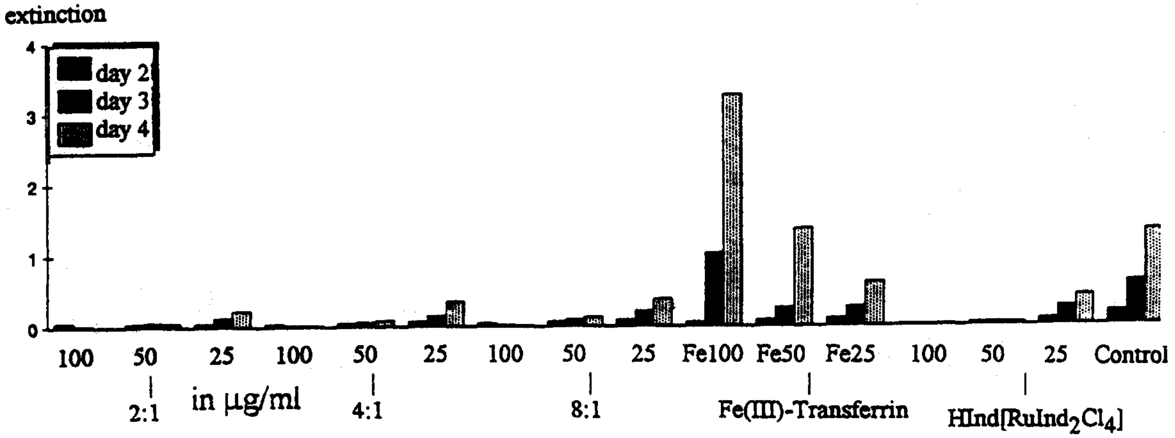

**Figure 1B: Comparison of the inhibitory effect of the free complex HInd[$RuInd_2Cl_4$] and its apotransferrin-bound forms in the human colon cancer cell line SW707 using the microculture tetrazolium test (MTT)**

Different amounts of HInd[$RuInd_2Cl_4$] (2,4 and 8 equivalents) were bound to apotransferrin. 100, 50, 25 represent 100, 50 or 25 g/ml of HInd[$RuInd_2Cl_4$]. A100, A50, A25 are comparative amounts of pure apotransferrin corresponding to the amounts of the protein used to prepare the protein-bound forms of HInd[$RuInd_2Cl_4$].

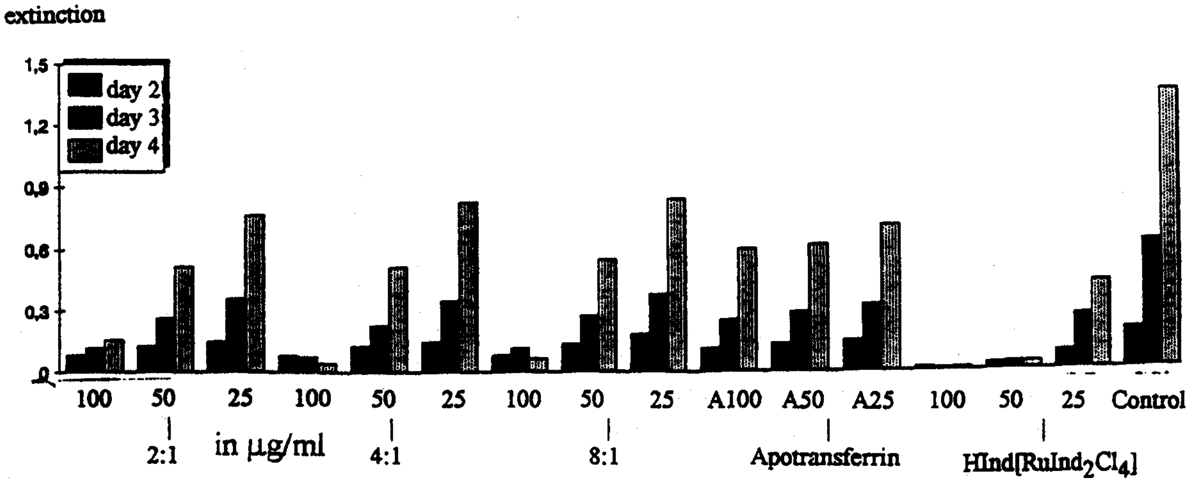

Figure 2A: Comparison of the inhibitory effect of the free complex $HIm[RuIm_2Cl_4]$ and its $Fe(III)_2$-transferrin-bound forms in the human colon cancer cell line SW707 using the microculture tetrazolium test (MTT).

Different amounts of $HIm[RuIm_2Cl_4]$ (2,4 and 8 equivalents) were bound to $Fe(III)_2$-transferrin. 100, 50, 25 represent 100, 50 or 25 g/ml of $HIm[RuIm_2Cl_4]$. Fe100, Fe50, Fe25 are comparative amounts of pure $Fe(III)_2$-transferrin corresponding to the amounts of the protein used to prepare the protein-bound forms of $HIm[RuIm_2Cl_4]$

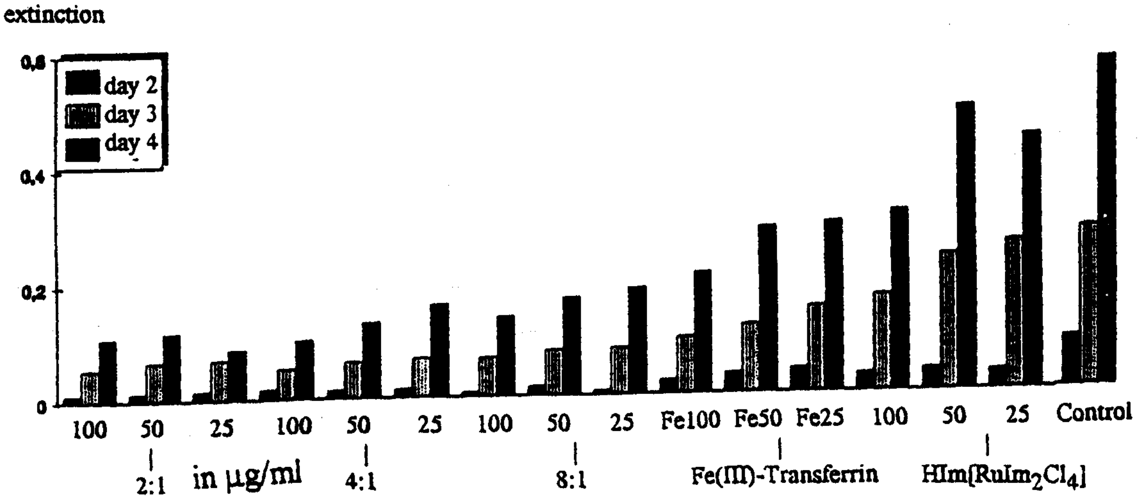

Figure 2B: Comparison of the inhibitory effect of the free complex $HIm[RuIm_2Cl_4]$ and its apotransferrin-bound forms in the human colon cancer cell line SW707 using the microculture tetrazolium test (MTT).

Different amounts of $HIm[RuIm_2Cl_4]$ (2,4 and 8 equivalents) were bound to apotransferrin. 100, 50, 25 represent 100, 50 or 25 g/ml of $HIm[RuIm_2Cl_4]$. A100, A50, A25 are comparative amounts of pure apotransferrin corresponding to the amounts of the protein used to prepare the protein-bound forms of $HIm[RuIm_2Cl_4]$.

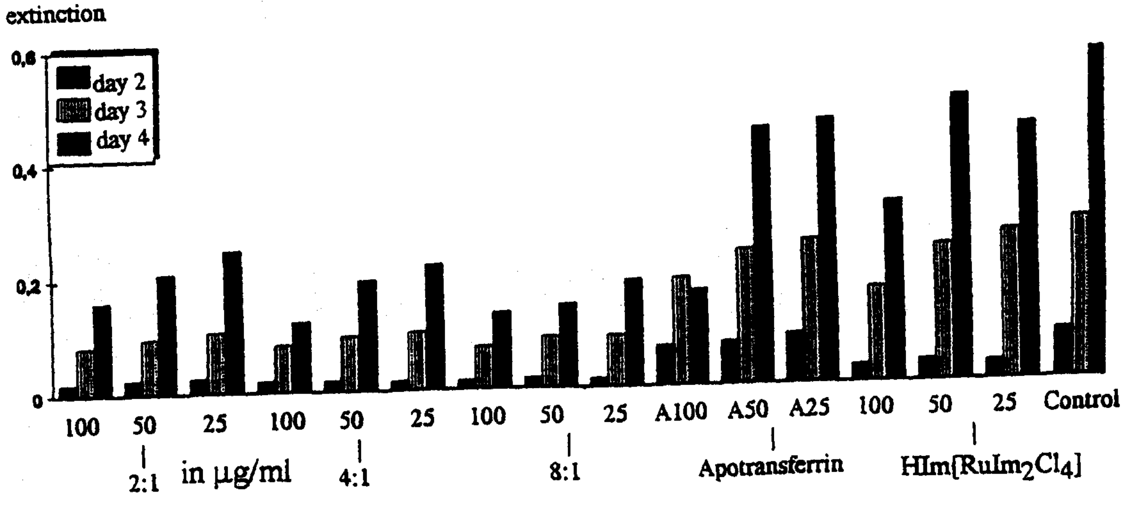

In contrast, the apotransferrin-bound forms of the second complex, HIm[$RuIm_2Cl_4$], exhibit a stronger inhibitory effect than the free complex, the protein-bound forms being about twice as active as the free complex (Figure 2B). Similar results were obtained for the transferrin-bound form (Figure 2A), the protein-bound form being two to three times more effective than the free complex. Surprisingly, transferrin on its own did not stimulate cell growth in this experiment perhaps due to the less pronounced growth of the tumor cells which was slower than in the experiments with HInd[$RuInd_2Cl_4$].

In both experiments the amount of complex bound to the proteins (2, 4, or 8 equivalents) did not have a significant effect on antiproliferative efficacy.

### Discussion

It has been shown recently that the most promising antitumour Ru(III) complex, HInd[$RuInd_2Cl_4$], which is far less toxic in long term application than the imidazole analogue, binds within a few minutes to the serum proteins albumin and transferrin (Kratz et al., 1992). This result suggests that apotransferrin or transferrin could act as a natural carrier of the drug to the tumor tissues owing to the high affinity between this metal transport protein and the large number of transferrin receptors on the surface of tumor cells [Brock, 1985). There is meanwhile a considerable amount of evidence available which demonstrates hat tumours in animals and man exhibit a very high, "parasitic" uptake of iron(III) when they are fast growing. Aulbert has reviewed the literature on this phenomenon (Aulbert, 1986) and together with his investigations demonstrated that tumour cells exhibit a high amount of transferrin receptors by which they sequester iron. Especially fast growing tumour cells show a much higher uptake of iron-transferrin than the normal surrounding tissue of the affected organ. For these reasons, it is therefore tempting to exploit the transferrin cycle as a "natural" route for the selective delivery of cytostatic drugs to cancer cells.

Transferrin is the iron-carrying protein in the serum of vertebrates. It is a β-glycoprotein with a molecular weight of 80 000 and is present in human serum at a concentration of 2.5-3.5 mg/ml. The iron-free transferrin is called apotransferrin and can bind two equivalents of Fe(III) in the N- and C-terminal lobes of the protein upon which the protein undergoes a conformational change.

When encountering the cell surface, iron-bound transferrin binds to a specific cell surface receptor (transferrin receptor). The next step is internalisation of the transferrin-receptor complex by endocytosis, followed by removal of iron from transferrin with the aid of intracellular chelates and involves fusion with endosomes, where the pH is lower (pH = 5); finally the apotransferrin is returned to the extracellular medium.

If the ruthenium complexes are being transported by transferrin or apotransferrin to the tumour cells, they have to be released again to exert their antitumour effect after the protein/complex-adduct has entered the tumour cell. The release should occur at low pH-values (pH = 5) with the aid of chelates. We have previously shown that both Ru(III) complexes can be released from apotransferrin or transferrin at pH 4-5 in the presence of citric acid or ATP (Kratz et al., 1994), presumably in a form in which the chlorides of the original complex are substituted by citrate and ATP respectively, so that the cellular uptake of the Ru(III) complexes could follow the transferrin path.

Our cell culture experiments demonstrate that the transferrin-bound forms of both complexes exhibit high antiproliferative activity which even exceeds that of the free complex.

The results for apotransferrin-bound forms, however, show a different picture: the apotransferrin-bound form of HInd[RuInd$_2$Cl$_4$] is not as active as the respective free complex, but the bound form of HIm[RuIm$_2$Cl$_4$] is. This difference could possibly be explained by the larger change in the protein conformation when HIm[RuIm$_2$Cl$_4$] binds to apotransferrin due to the smaller N-heterocycle. This change in the conformation is then similar to that that which takes place when iron(III) is bound then, so that the high affinity between the transferrin receptor and transferrin-bound form of the complex is retained.

Although we have not demonstrated that the Ru(III) complexes are released inside the cell, the transferrin-bound forms of both complexes and the apotransferrin-bound form of HIm[RuIm$_2$Cl$_4$] retain their antiproliferative effect. Our results therefore shed light on the discrepancies observed between the *in vitro* $IC_{50}$ values of HInd[RuInd$_2$Cl$_4$] (110 mg/ml) and the dosages active *in vivo* (13.0 mg/kg). Tissue levels as high as the $IC_{50}$ value can only be reached for short periods of time at this in vivo dosage or by tissue accumulation which could be achieved by transferrin acting as a carrier of the Ru(III) complex.

We are therefore planning animal experiments to strengthen our working hypothesis.

*Acknowledgements:* The support of the Dr. Mildred-Scheel Stiftung der Deutschen Krebshilfe, Bonn, FRG, is gratefully acknowledged.

**References**

Aulbert E, (1986): Transferrinmangelanaemie bei malignen Tumorerkrankungen, Georg Thieme Verlag Stuttgart, New York.

Berger MR, Garzon FT, Keppler BK, Schmähl D (1989) Efficacy of new ruthenium complexes against chemically induced autochthonous colorectal carcinoma in rats. *Anticancer Res 9*, 761-766

Brock JH (1985): Transferrins, 183-263, *in Metalloproteins*, *Part 2*, ed. P.M. Harrison, VCH, Weinheim.

Clarke MJ, (1989): Ruthenium chemistry pertaining to the design of anticancer agents, *Progress in Clinical Biochemistry and Medicine, Vol. 10*, Springer-Verlag Berlin Heidelberg, 25-39.

Galeano A, Berger MR, Keppler BK, (1992): *Arzneim.-Forsch/Drug Res. 42*, (6), 821-824.

Harris DC and Aisen P, (1989): Physical Biochemistry of the Transferrins, in *Iron Carriers and Iron Proteins, Physical Bioinorganic Chemistry Series*, ed. by T.M. Loehr, VCH Weinheim, 239 -352.

Keppler BK, Henn M, Juhl UM, Berger MR, Niebl R, and Wagner FE, (1989): New ruthenium complexes for the treatment of cancer, *Prog. Clin. Biochem. Med., vol.10*, 41-69, Springer Verlag, Berlin Heidelberg.

Kratz F, Mulinacci N, Messori L, Bertini I, Keppler BK, (1992): Interactions of antitumour Ru(III) complexes with serum proteins, *Metal Ions in Biology and Medicine, vol.2,* John Libbey Limited, 69-74.

Kratz F (1993): Interactions of antitumour metal complexes with serum proteins. Perspectives for anticancer drug development., in *Metal Complexes in Cancer Chemotherapy*, ed. B.K. Keppler, Verlag-Chemie, 392-429.

Kratz F, Hartmann M, Keppler BK and Messori L (1994a): The binding properties of two antitumor ruthenium(III) complexes to apotransferrin, *J. Biol. Chem*, 269(4), 2581-2588.

Kratz F, Keppler BK, Messori L, Smith L, Baker EN (1994b): Protein-binding properties of two antitumour Ru(III) complexes to human apotransferrin and apolactoferrin, *Metal-based Drugs*, 1, ed. M. Gielen, *in press.*

Ward SG and Taylor RC (1988) in *Metal based Antitumor Drugs* (Gielen MF, ed.) Freund Publishing House Ltd., chapter 1, 1-54.

**Received: November 6, 1995 - Accepted: November 24, 1995 - Received in revised camera-ready format: December 19, 1995**

